# *Fusobacterium nucleatum* promotes colorectal cancer cells adhesion to endothelial cells and facilitates extravasation and metastasis by inducing ALPK1/NF-κB/ICAM1 axis

**DOI:** 10.1080/19490976.2022.2038852

**Published:** 2022-02-27

**Authors:** Ying Zhang, Lu Zhang, Sheng Zheng, Mengjie Li, Chaochao Xu, Dingjiacheng Jia, Yadong Qi, Tongyao Hou, Lan Wang, Boya Wang, Aiqing Li, Shujie Chen, Jianmin Si, Wei Zhuo

**Affiliations:** a Department of Gastroenterology, Sir Run Run Shaw Hospital, Zhejiang University School of Medicine, Hangzhou, China; b Department of Cell Biology and Department of Gastroenterology, Sir Run Run Shaw Hospital, Zhejiang University School of Medicine, Hangzhou, China; c Cancer Center, Zhejiang University, Hangzhou, China; d Institute of Gastroenterology, Zhejiang University, Hangzhou, China; e Department of Gastroenterology, Second Affiliated Hospital of Zhejiang University School of Medicine, Hangzhou, China; f Department of Pharmacy, Sir Run Run Shaw Hospital, Zhejiang University School of Medicine, Hangzhou, China

**Keywords:** Gut microbes, adhesion, ICAM1, ALPK1, colorectal cancer metastasis

## Abstract

Metastasis is the leading cause of death for colorectal cancer (CRC) patients, and the spreading tumor cells adhesion to endothelial cells is a critical step for extravasation and further distant metastasis. Previous studies have documented the important roles of gut microbiota-host interactions in the CRC malignancy, and *Fusobacterium nucleatum* (*F. nucleatum*) was reported to increase proliferation and invasive activities of CRC cells. However, the potential functions and underlying mechanisms of *F. nucleatum* in the interactions between CRC cells and endothelial cells and subsequent extravasation remain unclear. Here, we uncovered that *F. nucleatum* enhanced the adhesion of CRC cells to endothelial cells, promoted extravasation and metastasis by inducing ICAM1 expression. Mechanistically, we identified that *F. nucleatum* induced a new pattern recognition receptor ALPK1 to activate NF-κB pathway, resulting in the upregulation of ICAM1. Interestingly, the abundance of *F. nucleatum* in tumor tissues of CRC patients was positively associated with the expression levels of ALPK1 and ICAM1. Moreover, high expression of ALPK1 or ICAM1 was significantly associated with a shorter overall survival time of CRC patients. This study provides a new insight into the role of gut microbiota in engaging into the distant metastasis of CRC cells.

## Introduction

Colorectal cancer (CRC) is the fourth most commonly diagnosed cancer worldwide and the second most frequent cause of death closely following lung cancer.^1^ Cancer metastasis, a major clinical problem of human cancer, is responsible for most colorectal cancer patient mortality.^2^ Thus, it is important to elucidate the underlying mechanisms of metastasis in CRC patients.

During metastasis, cancer cells leave the original tumor organ and migrate to the target metastasis organs through a process that involves intravasation, adhesion and extravasation, among which the adhesion to endothelial cells and trans-endothelial invasion of tumor cells are key steps in the metastatic process.^3,4^ Indeed, changes in the expression or functions of cell adhesion molecules have been implicated in all steps of tumor progression. Cell adhesion molecules belonging to the immunoglobulin superfamily commonly play critical and necessary roles in metastasis,^5–7^ among which intercellular adhesion molecule 1 (ICAM1) is a well-known transmembrane glycoprotein involved in cell-cell direct interactions.^7^ The interaction between ICAM1 and its specific ligand could facilitate the adhesion of cancer cells to the vascular endothelium and subsequently in the promotion of metastasis. Importantly, the expression of ICAM1 was positively correlated with cancer progression and metastasis.^8–10^

Multiple studies have suggested a potential link between *Fusobacterium nucleatum* (*F. nucleatum*) and colorectal cancer.^11,12^ *F. nucleatum* is a Gram-negative anaerobic oncogenic bacterium enriched in human colorectal carcinoma compared with adjacent normal tissues.^13–15^ The overabundance of *F. nucleatum* in CRC tissues has been reported to promote CRC cell proliferation, recurrence and chemoresistance.^16–18^ However, the potential effects and underlying mechanisms of *F. nucleatum* in the interactions between CRC cells and endothelial cells and subsequent extravasation in CRC metastasis remain largely unknown.

NF-κB, initially established as an important transcription factor, has been demonstrated to participate in inflammation-associated colorectal cancer.^19^ It is reported that *F. nucleatum*-mediated infection leads to the activation of NF-κB pathway via the suggested membrane receptor-TLR4/MYD88 cascade.^17^ However, previous studies confirmed the invasive potential of *F. nucleatum* into CRC cells undergoing a non-obligate intracellular life stage, and it could be detected in CRC metastases of liver and lymph nodes,^20,21^ indicating that there may be another pathway to activate NF-κB in *F. nucleatum*-infected CRC cells. Interestingly, a recent study reported that alpha-kinase 1 (ALPK1), a member of the α-kinase family in cytoplasm, functions as a cytosolic innate immune receptor for bacterial adenosine diphosphate-heptose (ADP-Hep), which is present in all Gram-negative and some Gram-positive bacteria.^22,23^ It is reported that ALPK1 plays a crucial role in the activation of NF-κB signaling and subsequent production of pro-inflammatory cytokines and chemokines through the stimulation of its kinase domain.^24,25^ Therefore, we wondered the potential effects and mechanisms of ALPK1 in *F. nucleatum*-mediated CRC metastasis.

For the first time, our study proposed a *F. nucleatum*-induced cell-cell interaction model by which *F. nucleatum* treatment promoted CRC cells adhesion to endothelial cells, facilitated extravasation and metastasis through upregulating ICAM1 expression in CRC cells. Mechanistically, we uncovered that *F. nucleatum* upregulated the expression of ICAM1 by activating ALPK1/NF-κB pathway in CRC cells.

## Results

### *F.* *nucleatum* promotes CRC cells adhesion to endothelial cells and facilitates extravasation by upregulating ICAM1.

**Figure 1.**
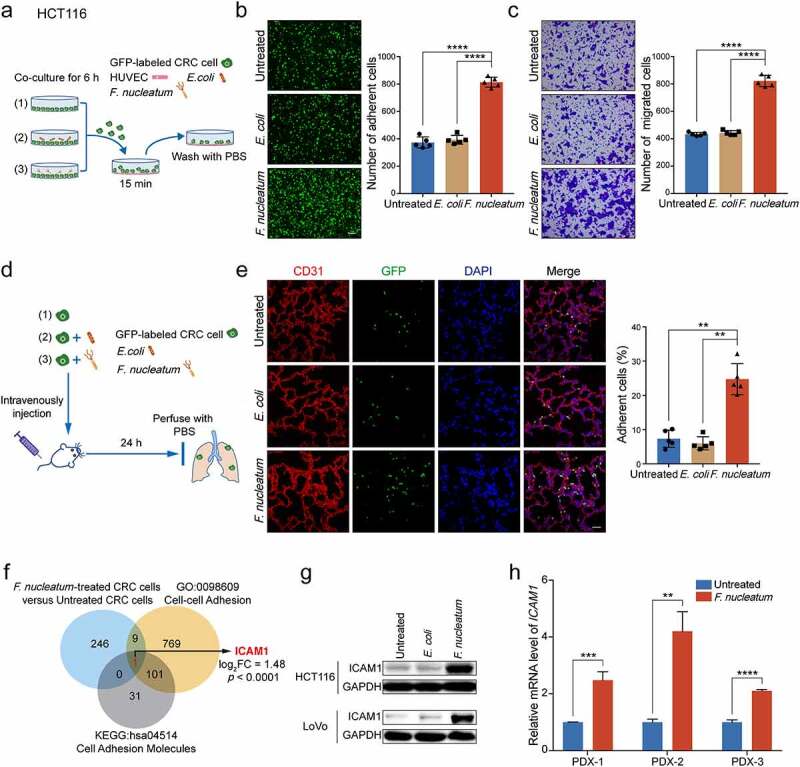
***F. nucleatum* promotes CRC cells adhesion to endothelial cells and facilitates extravasation by upregulating ICAM1**. (a) Schematic illustration of adhesion assay *in vitro*. (b) GFP-labeled HCT116 cells pretreated with *F. nucleatum, E. coli* or PBS control for 6 hours were subjected to adhesion assay. Representative images of adherent cells were shown (left) and the adherent cells were quantified by counting in five fields (right). Scale bar, 100 μm. (c) HCT116 cells were pretreated with *F. nucleatum, E. coli* or PBS control for 6 hours and subjected to migration assay. The migrated cells were quantified at 18 hours by counting in five fields. Scale bar, 100 μm. (d) Schematic illustration of cancer cell extravasation model *in vivo*. (e) The colonized GFP-labeled HCT116 cells in mice lung tissues were quantified by immunofluorescence assay, in which lung sections were stained with a marker of vascular endothelial cells CD31 (red), and the nuclei were counterstained with DAPI (blue). Scale bar, 100 μm. (f) 256 genes with significant mRNA upregulation (fold change > 2, *p*.adj < 0.05) in RNA-sequencing, 880 genes in the GO class of cell-cell adhesion (GO: 0098609), and 133 genes in the KEGG pathway of cell adhesion molecules (hsa04514) with key functions were subjected to Venn diagram analysis. Venn diagram showing the overlapping genes. (g) Western blot analysis was performed to detect the protein levels of ICAM1 in HCT116 cells and LoVo cells treated with *F. nucleatum, E. coli* or PBS control. (h) Quantitative RT-PCR analysis of *ICAM1* in PDX tumor tissues treated with *F. nucleatum* or PBS control. Data are shown as mean ± SD. ** *P*< .01, *** *P* < .001, **** *P* < .0001, Student’s t test (b, c, h), Mann-Whitney test (e).

When we study the role of gut microbiota in CRC cell malignancy, an interesting phenomenon during culturing CRC cells attracted our attention that *F. nucleatum* treatment, but not *Escherichia coli* (*E. coli*) or phosphate buffer saline (PBS) control, may increase cell-cell interaction ability of CRC cells. As we know, the spreading tumor cells adhesion to endothelial cells is a critical step for extravasation and further distant metastasis. It is possible that the *F. nucleatum*-induced adhesion ability of CRC cells may facilitate its interaction with endothelial cells and extravasation. To verify our hypothesis, we designed a tumor cell-endothelial cell interaction model. Human Umbilical Vein Endothelial Cells (HUVECs) were seeded in culture plates until full confluence. Then GFP-labeled HCT116 cells pretreated with *F. nucleatum* (*F. nucleatum* strain 25586), *E. coli* or PBS control were added onto the HUVECs monolayers. The unbound tumor cells were washed away after 15 minutes and the adherent HCT116 cells were analyzed (Figure 1(a)). *F. nucleatum* treatment strongly enhanced the adhesion of HCT116 cells to HUVECs compared with *E. coli* or PBS control (Figure 1(b)). Meanwhile, in concordance with our previous findings,^26^ *F. nucleatum* treatment dramatically promoted cell migration of both HCT116 cells (Figure 1(c)) and LoVo cells (Supplementary Figure 1a). In addition, another *F. nucleatum* strain 10953 was applied to assess whether this is a general property of *F. nucleatum. F. nucleatum* strain 10953 treatment, similar with *F. nucleatum* strain 25586, obviously promoted CRC cell-endothelial cell adhesion and CRC cells migration (Supplementary Figure 1b-c), suggesting that this may be a general property of *F. nucleatum*. Interestingly, when we employed a Gram-negative bacteria *Akkermansia muciniphila* (*A. muciniphila*)^27^ and a Gram-positive bacteria *Bifidobacterium adolescentis* (*B. adolescentis*)^28^ to identify the specific phenotype of *F. nucleatum*, we found that *F. nucleatum* treatment dramatically promoted CRC cells adhesion to endothelial cells, as well as the migration ability, but not *A. muciniphila* or *B. adolescentis* (Supplementary Figure 1d-e).

To confirm these *in vitro* results, we intravenously injected *F. nucleatum*-treated GFP-labeled HCT116 cells into SCID mice. The pulmonary blood vessels were perfused with PBS after 24 hours and the colonized HCT116 cells in lungs were analyzed (Figure 1(d)). *F. nucleatum* treatment robustly increased the trans-endothelial invasion and colonization of HCT116 cells compared with *E. coli* or PBS treatment (Figure 1(e)).

To understand the underlying mechanisms of *F. nucleatum*-induced intercellular adhesion ability of CRC cells, we performed RNA-sequencing in HCT116 cells with or without *F. nucleatum* treatment (GSE171611). As shown in Figure 1(f), we identified gene *ICAM1* overlapping among *F. nucleatum*-upregulated genes (log_2_ fold change > 1, *p*.adj < 0.05), the GO class of cell-cell adhesion and the KEGG pathway of cell adhesion molecules. To confirm the profiling results, we detected the protein expression of ICAM1 in *F. nucleatum-*treated CRC cells and observed that ICAM1 was indeed significantly increased in *F. nucleatum-*treated HCT116 cells and LoVo cells (Figure 1(g)). Importantly, we established patient-derived xenograft (PDX) model with tumor tissues generated from CRC patients. All of the PDX tumor tissues from three patients treated with *F. nucleatum ex vivo* showed a significant upregulation of ICAM1 levels (Figure 1(h)).

### ICAM1 is involved in *F. nucleatum*-induced CRC cells adhesion to endothelial cells and migration *in* *vitro*.

**Figure 2.**
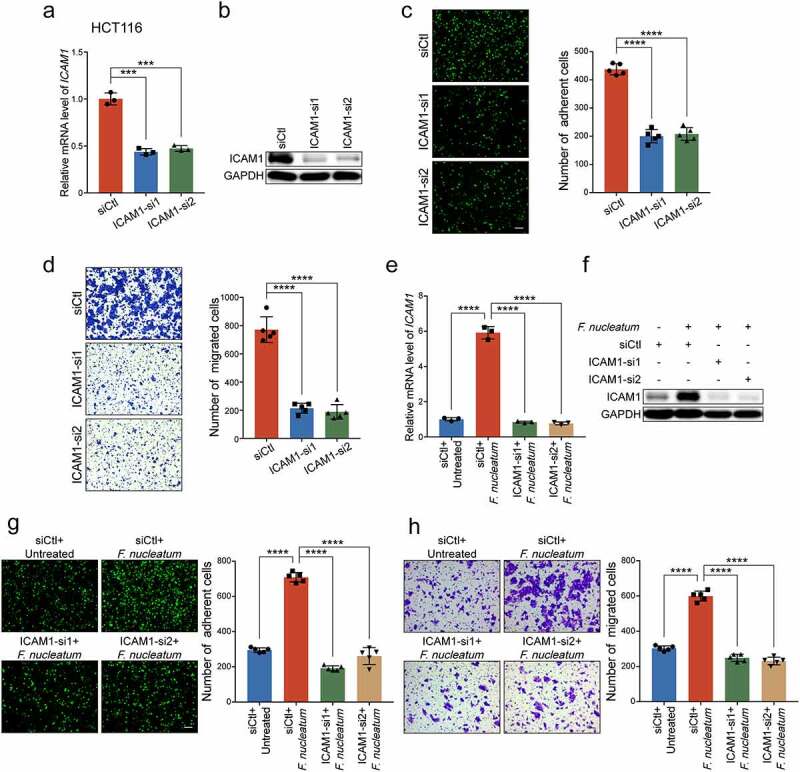
**ICAM1 is involved in *F. nucleatum*-induced CRC cells adhesion to endothelial cells and migration *in vitro***. (a-b) Quantitative RT-PCR (a) and Western blot analysis (b) of ICAM1 were performed in HCT116 cells transfected with two siRNAs targeting ICAM1 or control siRNAs. (c-d) Adhesion assay (c) and migration assay (d) of HCT116 cells transfected with the indicated siRNAs. The migrated cells were observed at 24 hours. Scale bar, 100 μm. (e-f) Quantitative RT-PCR (e) and Western blot analysis (f) were performed in HCT116 cells. They were transfected with the indicated siRNAs, and then co-cultured with *F. nucleatum* or PBS. (g-h) HCT116 cells transfected with the indicated siRNAs were co-cultured with *F. nucleatum*, and subjected to adhesion assay (g) and migration assay (h). The migrated cells were observed at 18 hours. Scale bar, 100 μm. Data are shown as mean ± SD. *** *P* < .001, **** *P* < .0001, by Student’s t test.

To further understand the functions of ICAM1 in CRC progression, we silenced ICAM1 in HCT116 cells using two specific siRNAs (Figure 2(a**-b)**). Knockdown of ICAM1 dramatically inhibited the adhesion of HCT116 cells to HUVECs (Figure 2(c)). Meanwhile, as shown in Figure 2(d), ICAM1 silencing showed remarkable tumor migration suppressive effects. Similar results were also observed in LoVo cells (Supplementary Figure 2a-c).

To further determine whether *F. nucleatum* exerted oncogenic functions in an ICAM1 dependent manner, we performed ICAM1 loss of function assays in CRC cells. The upregulated mRNA and protein levels of ICAM1 in *F. nucleatum-*treated CRC cells were significantly decreased when we silenced ICAM1 in HCT116 cells (Figure 2(e**-f)**) and LoVo cells (Supplementary Figure 2d-e). As expected, ICAM1 knockdown abrogated the promotion of *F. nucleatum*-mediated HCT116 cells adhesion to endothelial cells (Figure 2(g)). Moreover, silencing of ICAM1 significantly reversed the increased cell migration ability induced by *F. nucleatum* (Figure 2(h), Supplementary Figure 2f), highlighting the important role of ICAM1 in sustaining the functions mediated by *F. nucleatum*. Taken together, we suggest that ICAM1 is responsible for CRC cells adhesion to endothelial cells and migration induced by *F. nucleatum in vitro*.

### ICAM1 is involved in *F. nucleatum*-mediated CRC cells extravasation and metastasis *in* *vivo*.

**Figure 3.**
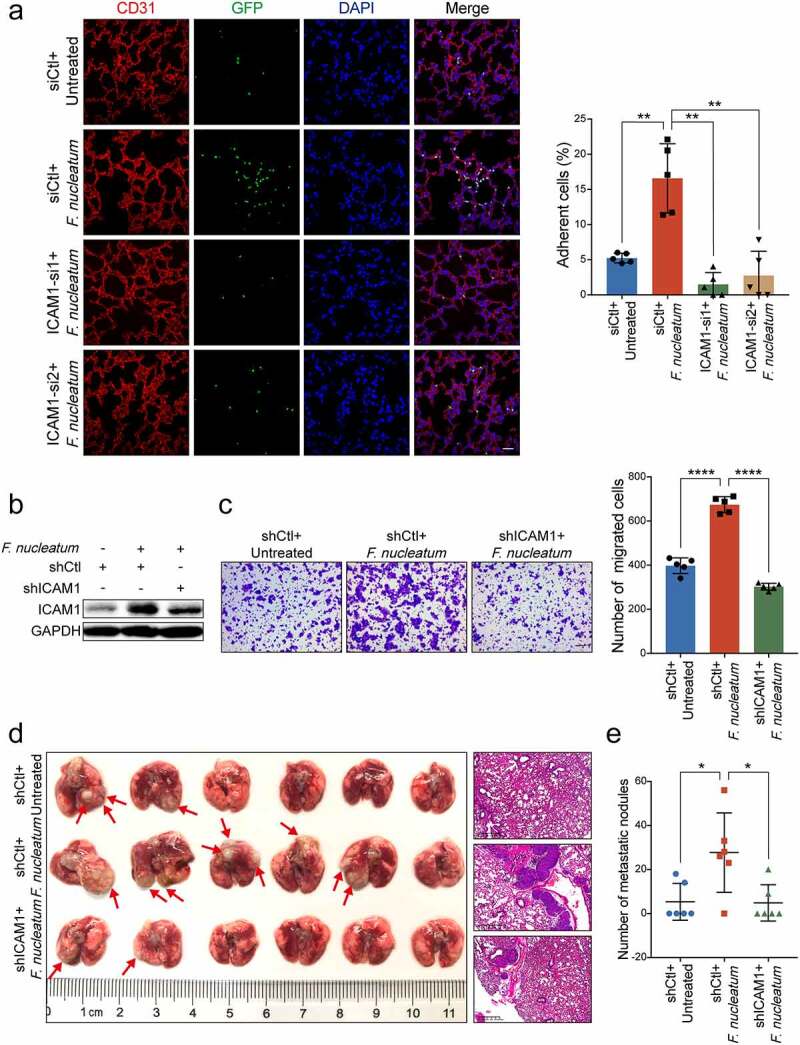
**ICAM1 is involved in *F. nucleatum*-mediated CRC cells extravasation and metastasis *in vivo***. (a) GFP-labeled HCT116 cancer cells transfected with two siRNAs targeting ICAM1 were co-cultured with *F. nucleatum* or PBS, and subjected to extravasation model *in vivo*. The colonized HCT116 cells in lung tissues were measured by immunofluorescence assay. Scale bar, 100 μm. (b-c) HCT116 cells stably infected with lentivirus-based ICAM1 shRNAs or control shRNAs were co-cultured with *F. nucleatum* or PBS control, and subjected to Western blot analysis (b) and migration assay (c). The migrated cells were quantified at 18 hours. Scale bar, 100 μm. (d-e) The ICAM1-knockdown HCT116 cells pretreated with *F. nucleatum* or PBS were tail-vein injected into nude mice to develop pulmonary metastases. Representative gross lungs and H&E stained lung sections were shown (d). Arrows indicated metastatic nodules. Pulmonary metastatic nodules per mice were quantified (e). Data are shown as mean ± SD. * *P* < .05, ** *P* < .01, **** *P* < .0001, Mann-Whitney test (a, e), Student’s t test (c).

To further assess the role of ICAM1 in *F. nucleatum*-mediated trans-endothelial invasion and colonization *in vivo*, we depleted ICAM1 expression in GFP-labeled HCT116 cells and treated cells with *F. nucleatum*. The cancer cell extravasation assay showed that *F. nucleatum* dramatically promoted HCT116 cells extravasation, while knockdown of ICAM1 obviously reversed the trans-endothelial invasion and colonization (Figure 3(a)). In addition, we established stable ICAM1-knockdown HCT116 cells by lentivirus-based shRNAs (Figure 3(b)). In concordance with our previous findings, knockdown of ICAM1 significantly diminished the cell migration ability induced by *F. nucleatum* (Figure 3(c)). We intravenously inoculated mice with the indicated cells to measure pulmonary metastases. Data from the gross image and H&E staining of lungs showed that *F. nucleatum* significantly increased the number of metastatic lesions, whereas knockdown of ICAM1 in HCT116 cells notably diminished *F. nucleatum*-induced lung metastasis (Figure 3(d**-e)**). Taken together, these data suggest that *F. nucleatum* promotes extravasation and metastasis of CRC cells via the upregulation of ICAM1 *in vivo*.

### *F.* *nucleatum* upregulates ICAM1 expression through the activation of NF-κB signaling pathway.

**Figure 4.**
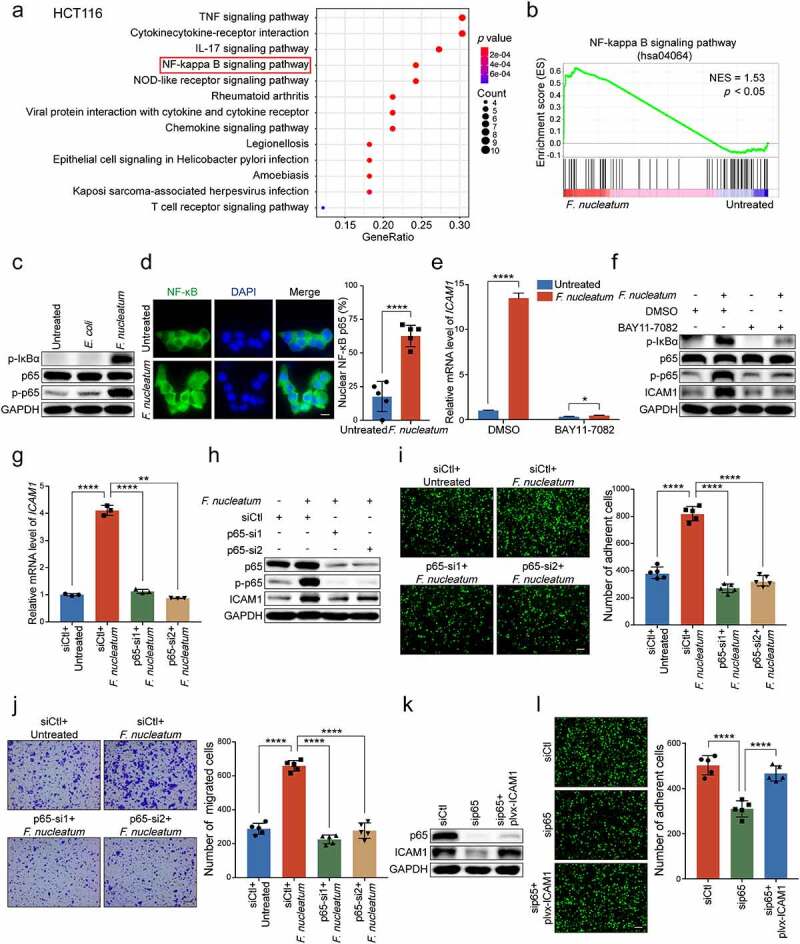
***F.******nucleatum* upregulates ICAM1 expression through the activation of NF-κB signaling pathway.** (a) KEGG pathway analysis of a total number of 503 genes upregulated with significant difference in RNA-sequencing. (b) GSEA showed the differentially expressed gene cluster related to NF-κB signaling pathway in HCT116 cells with or without *F.* *nucleatum* treatment. (c) Western blot analysis of phospho-IκBα, NF-κB subunit p65 and phospho-p65 in HCT116 cells treated with *F.* *nucleatum*, *E.* *coli* or PBS control. (d) Immunofluorescence assay of NF-κB p65 distribution in the indicated HCT116 cells. Cells were stained with specific antibody against p65 (green), and the nuclei were counterstained with DAPI (blue). Scale bar, 20 μm. (e-f) HCT116 cells were co-cultured with *F.* *nucleatum* or PBS control, and then treated with NF-κB inhibitor, BAY11-7082. Quantitative RT-PCR (e) and Western blot analysis (f) were performed. (g-h) HCT116 cells were transfected with two siRNAs targeting p65, and then co-cultured with *F.* *nucleatum* or PBS control. Quantitative RT-PCR (g) and Western blot analysis (h) were performed. (i-j) Adhesion assay (i) and migration assay (j) of HCT116 cells with indicated treatments. The migrated cells were observed at 18 hours. Scale bar, 100 μm. (k-l) P65-depleted HCT116 cells transfected with the indicated plasmids were applied for Western blot analysis (k) and adhesion assay (l). Scale bar, 100 μm. Data are shown as mean ± SD. * *P* < .05, ** *P* < .01, **** *P* < .0001, by Student’s t test.

We next tried to investigate the underlying mechanisms for *F.* *nucleatum* upregulating the expression of ICAM1. KEGG pathway analysis and Gene Set Enrichment Analysis (GSEA) of the RNA-sequencing results suggested that NF-κB signaling pathway was activated by *F.* *nucleatum* infection (Figure 4(a**-b)**). Western blot analysis confirmed that the phosphorylation levels of NF-κB subunit p65 was substantially upregulated, as well as the phosphorylation levels of NF-κB inhibitor IκBα in HCT116 cells (Figure 4(c)) and LoVo cells (Supplementary Figure 3a) when treated with *F.* *nucleatum*, but not *E.* *coli* or PBS. Immunofluorescence assay also showed an increase in the nuclear localization of NF-κB p65 in cells with *F.* *nucleatum* treatment (Figure 4(d), Supplementary Figure 3b). Collectively, these data indicate that NF-κB signaling is activated in CRC cells by *F.* *nucleatum*.

We were curious whether the activated NF-κB pathway was involved in the regulation of ICAM1. BAY11-7082, an inhibitor of NF-κB, was employed to treat CRC cells when infected with *F.* *nucleatum*. Both quantitative RT-PCR and Western blot analysis showed that the upregulation of ICAM1 induced by *F.* *nucleatum* infection was notably attenuated when HCT116 cells (Figure 4(e**-f)**) and LoVo cells (Supplementary Figure 3c-d) were treated with NF-κB inhibitor. Moreover, the knockdown of p65 also inhibited *F.* *nucleatum*-induced ICAM1 upregulation in HCT116 cells (Figure 4(g**-h**)) and LoVo cells (Supplementary Figure 3e-f). These results suggest that the upregulation of ICAM1 in CRC cells is due to the activation of NF-κB signaling by *F.* *nucleatum*.

As expected, the knockdown of p65 in HCT116 cells significantly decreased the promotion of *F.* *nucleatum*-mediated adhesion to endothelial cells, as well as the migration ability (Figure 4(i**-j)**). Similar results were also observed in LoVo cells (Supplementary Figure 3g). Moreover, we reintroduced ICAM1 levels in p65-depleted CRC cells and found that ectopic expression of ICAM1 could rescue the inhibitory effects of p65 silencing on cell adhesion and migration (Figure 4(k**-l**), Supplementary Figure 3h-i).

Taken together, these results indicate that *F.* *nucleatum* treatment upregulates ICAM1 through activating NF-κB signaling pathway.

### ALPK1 mediates NF-κB-dependent responses to *F.* *nucleatum* infection.

**Figure 5.**
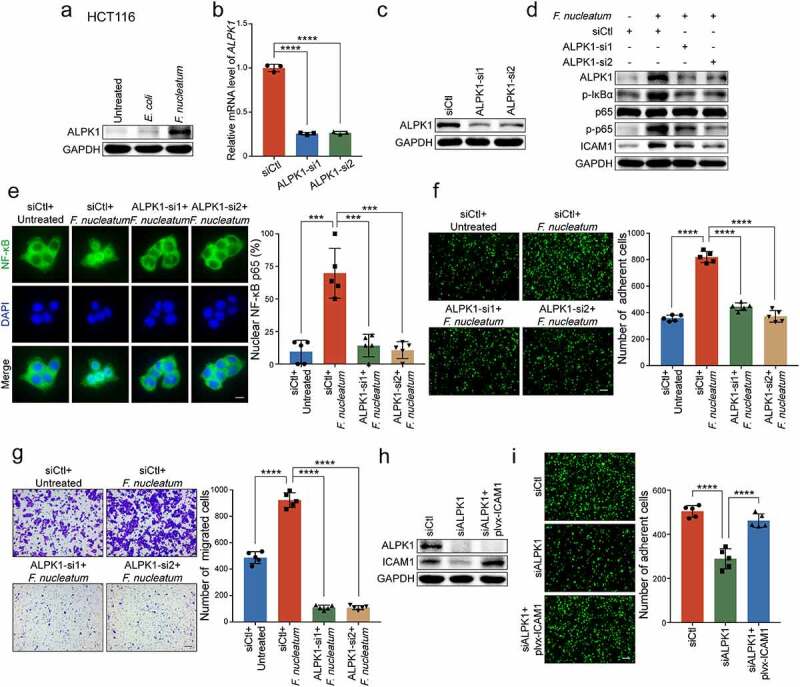
**ALPK1 mediates NF-κB-dependent responses to *F.*** ***nucleatum* infection.** (a) Western blot analysis of ALPK1 in HCT116 cells treated with *F.* *nucleatum*, *E.* *coli* or PBS control. (b-c) Quantitative RT-PCR (b) and Western blot analysis (c) of ALPK1 were performed in HCT116 cells transfected with two siRNAs targeting ALPK1 or control siRNAs. (d) HCT116 cells transfected with the indicated siRNAs were co-cultured with *F.* *nucleatum* or PBS control. Western blot analysis of ALPK1, phosphor-IκBα, p65, phospho-p65 and ICAM1 were performed. (e) The distribution of NF-κB p65 in the indicated cells was measured by immunofluorescence assay. Scale bar, 20 μm. (f-g) HCT116 cells transfected with the indicated siRNAs were co-cultured with *F.* *nucleatum* or PBS control. Adhesion assay (f) and migration assay (g) were performed. The migrated cells were observed at 18 hours. Scale bar, 100 μm. (h-i) ALPK1-depleted HCT116 cells transfected with the indicated plasmids were applied for Western blot analysis (h) and adhesion assay (i). Scale bar, 100 μm. Data are shown as mean ± SD. *** *P* < .001, **** *P* < .0001, by Student’s t test.

We next investigated the upstream regulators which could transmit signals to NF-κB pathway and mediate the functions of *F.* *nucleatum*. It is reported that Toll-like receptor family is involved in the system of gut microbiota-host interactions, and the TLR4/MYD88 cascade is activated in response to *F.* *nucleatum* intervention.^29^ However, we silenced TLR4 or MYD88 in *F.* *nucleatum*-treated HCT116 cells, and found that the knockdown of TLR4 or MYD88 could not reverse the promotion of adhesion ability induced by *F.* *nucleatum* (Supplementary Figure 4a). Intriguingly, previous studies have confirmed the distinct invasive potential of *F.* *nucleatum* into CRC cells undergoing a non-obligate intracellular life stage,^21^ and ALPK1 may function as a new pattern recognition receptor in the cytoplasm for ADP-Hep present in Gram-negative bacterial.^23^ Therefore, it is possible that *F.* *nucleatum* may activate NF-κB pathway through ALPK1 besides TLR4/MYD88 cascade in the cell membranes. To test this hypothesis, we treated HCT116 cells with *F.* *nucleatum*, *E.* *coli* or PBS control, and found that the protein level of ALPK1 was dramatically enhanced upon *F.* *nucleatum* treatment (Figure 5(a)). To further determine whether ALPK1 is involved in the regulation of *F.* *nucleatum*-mediated NF-κB activation, we depleted ALPK1 using two siRNAs targeting different regions of ALPK1 in HCT116 cells (Figure 5(b**-c)**). Western blot analysis showed that silencing of ALPK1 prevented NF-κB pathway from being activated by *F.* *nucleatum* infection (Figure 5(d)), which was also verified by immunofluorescence assay on the nuclear localization of NF-κB p65 (Figure 5(e)). Similar results were observed in LoVo cells (Supplementary Figure 4b-d). These results suggest that *F.* *nucleatum* activates NF-κB pathway through the upregulation of ALPK1.

Since *F.* *nucleatum* is able to activate ALPK1/NF-κB pathway, we wondered whether this pathway could be involved in the regulation of ICAM1. Interestingly, we observed that *F.* *nucleatum* failed to upregulate the expression of ICAM1 when ALPK1 was silenced in HCT116 cells (Figure 5(d)) and LoVo cells (Supplementary Figure 4c), highlighting the importance of ALPK1 in the upregulation of NF-κB-dependent ICAM1 by *F.* *nucleatum*. We then tried to determine whether ALPK1 mediated the functions of *F.* *nucleatum* in CRC progression. As expected, *F.* *nucleatum*-mediated CRC cells adhesion to endothelial cells was diminished when ALPK1 was knocked down in *F.* *nucleatum*-treated HCT116 cells (Figure 5(f)). Meanwhile, silencing of ALPK1 significantly impaired the *F.* *nucleatum*’s oncogenic effect on cell migration (Figure 5(g), Supplementary Figure 4e). Furthermore, we restored ICAM1 expression in ALPK1-depleted CRC cells and found that ectopic expression of ICAM1 significantly reversed the inhibitory effects of ALPK1 silencing on cell adhesion and migration (Figure 5(h**-i)**, Supplementary Figure 4f-g).

Together, these results suggest that *F.* *nucleatum* promotes CRC cell-endothelial cell adhesion and CRC cells migration through ALPK1/NF-κB/ICAM1 axis. To explore the specificity of *F.* *nucleatum* on ALPK1/NF-κB/ICAM1 axis, we further detected the protein levels of ALPK1, NF-κB signaling and ICAM1 in CRC cells with *A.* *muciniphila* or *B.* *adolescentis* treatment. Western blot analysis showed that *A.* *muciniphila* treatment, but not *B.* *adolescentis*, can slightly activate ALPK1/ NF-κB pathway (Supplementary Figure 4h-i). Interestingly, *F.* *nucleatum* treatment showed a much stronger stimulation than *A.* *muciniphila* on the ALPK1/NF-κB signaling, as well as the ICAM1 expression levels (Supplementary Figure 4h-i). Moreover, *F.* *nucleatum* strain 10953 could also notably activate ALPK1/NF-κB/ICAM1 signaling in HCT116 cells or LoVo cells comparable to the *F.* *nucleatum* strain 25586 (Supplementary Figure 4j-k).

### Overabundance of *F.* *nucleatum* correlates with high expressions of ALPK1 and ICAM1 in CRC patients and indicates poor clinical outcome.

**Figure 6.**
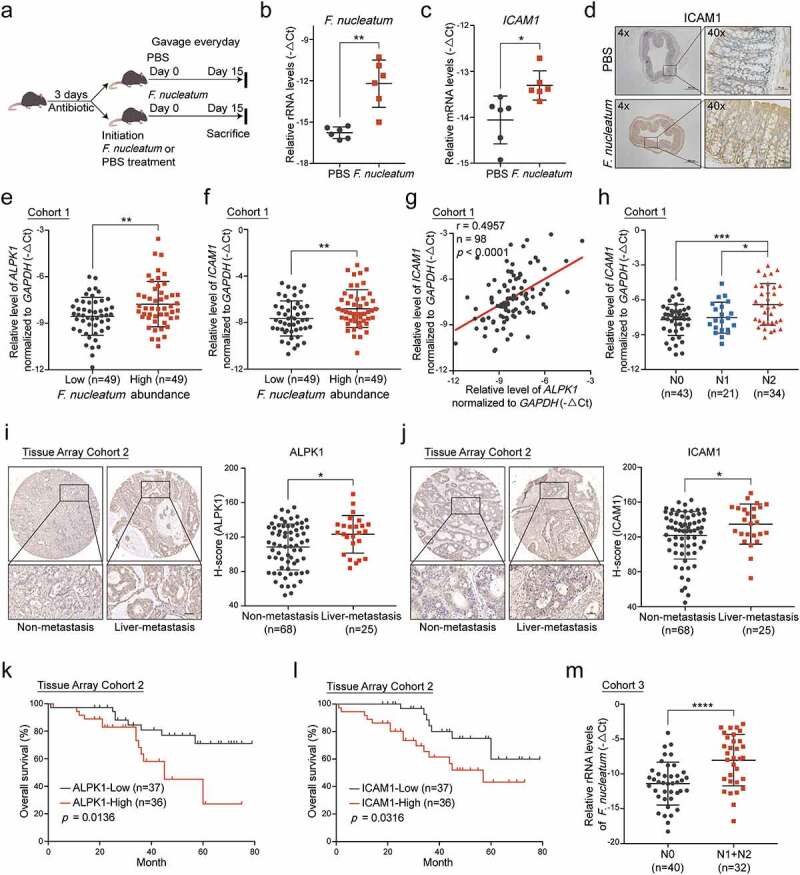
**Overabundance of *F.nucleatum* correlates with high expressions of ALPK1 and ICAM1 in CRC patients and indicates poor clinical outcome.** (a) C57BL/6 mice pretreated with antibiotics were administrated with *F.* *nucleatum* or PBS control everyday by gavage and sacrificed after the treatment at 15 days. (b) Quantitative RT-PCR analysis of the relative abundance of *F.* *nucleatum* in colon tissues from mice with the indicated treatment (n = 6). Data are normalized to *Universal Eubacteria 16S*. (c) Quantitative RT-PCR analysis of *ICAM1* mRNA expression in colon tissues from the indicated mice (n = 6). (d) Representative IHC images of ICAM1 protein expression in colon tissues from the indicated mice. (e-f) Quantitative RT-PCR analysis of *ALPK1* (e) and *ICAM1* (f) in Cohort 1 which was divided into two groups depends on the relative abundance of *F.* *nucleatum*. (g) The correlation between the relative mRNA levels of *ALPK1* and *ICAM1* in Cohort 1. (h) The relative mRNA levels of *ICAM1* among CRC patients with different lymph nodes metastasis stages in Cohort 1. (i-j) IHC analysis of ALPK1 (i) and ICAM1 (j) in Tissue Array Cohort 2. Representative photographs of IHC staining in CRC tissues with or without liver metastasis were shown (left), and the relative expression was quantified by H-score (right). Scale bar, 50 μm. (k-l) Kaplan-Meier survival curve of ALPK1 (k) and ICAM1 (l) expression of CRC patients in Tissue Array Cohort 2. (m) The relative abundance of *F.* *nucleatum* in the feces of CRC patients with positive lymph nodes metastasis (N1+N2, n = 32) or without metastasis (N0, n = 40) in Cohort 3. Data are shown as mean ± SD. * *P* < .05, ** *P* < .01, *** *P* < .001, **** *P* < .0001, Mann-Whitney test (b), Student’s t test (c, e, f, h, i, j, m), Linear Regression (g), Log-rank test (k, l).

Recent studies have revealed that the abundance of *F.* *nucleatum* is increased in colorectal cancer tissues versus matched normal tissues.^14,15^ To establish a high-*F.* *nucleatum* environment in the colon, we administrated *F.* *nucleatum* or PBS into C57BL/6 mice, which received antibiotics by gavage for 3 days beforehand (Figure 6(a)). After 15 days of treatment, mice were sacrificed and their colons were removed for the following experiments. Quantitative RT-PCR analysis revealed a significant increase of *F.* *nucleatum* in colon tissues with *F.* *nucleatum* treatment, indicating the model was successfully constructed (Figure 6(b)). Notably, *F.* *nucleatum* significantly increased ICAM1 expression (Figure 6(c-d)). Moreover, we established an AOM/DSS-induced CRC model accompanying with gavage treatment of *F.* *nucleatum*, *E.* *coli* or PBS control as previously described (Supplementary Figure 5a-b).^27,30^ Quantitative RT-PCR analysis revealed a significant upregulation of ALPK1 and ICAM1 levels in orthotopic tumor tissues from mice receiving *F.* *nucleatum* treatment compared with *E.* *coli* or PBS control (Supplementary Figure 5c-d). These results confirm that the overabundance of *F.* *nucleatum* in mouse colon tissues leads to the upregulation of ALPK1 and ICAM1 *in* *vivo*.

To further investigate the clinical significance of *F.* *nucleatum*/ALPK1/ICAM1, we quantified *F.* *nucleatum* and the expression of ALPK1 and ICAM1 with quantitative RT-PCR analysis in 98 paired fresh CRC and adjacent non-tumor tissues (Cohort 1). The relative amount of *F.* *nucleatum* was higher in CRC tissues compared with the non-tumor controls (Supplementary Figure 5e). We ranked these patients (n = 98) according to the levels of *F.* *nucleatum* in CRC tissues, and defined the top 1/2 patients as the *F.* *nucleatum-*high group (n = 49) and the bottom 1/2 patients as *F.* *nucleatum-*low group (n = 49) as previously described.^11^ Notably, we found the expression of ALPK1 and ICAM1 was significantly higher in the *F.* *nucleatum*-high group (Figure 6(e**-f)**). Similarly, there was a positive correlation between the relative abundance of *F.* *nucleatum* and ALPK1 levels in CRC tumor tissues (Supplementary Figure 5f), as well as the correlation between *F.* *nucleatum* and ICAM1 levels (Supplementary Figure 5g). Furthermore, the mRNA level of ICAM1 was positively associated with ALPK1 expression both in Cohort 1 and The Cancer Genome Atlas (TCGA) database (Figure 6(g), Supplementary Figure 5h). More importantly, tumor tissues from CRC patients with lymph nodes metastasis showed an upregulation of ICAM1 compared with tissues from non-metastasis patients, suggesting that ICAM1 was positively associated with advanced lymph nodes metastasis in CRC patients (Figure 6(h)). To further analyze the clinical significance of ALPK1 and ICAM1 in CRC patients with distant metastasis, we confirmed the expression of ALPK1 and ICAM1 in human CRC tissue array by IHC analysis (Tissue Array Cohort 2). Importantly, the high expression of ALPK1 or ICAM1 was significantly correlated with positive hepatic metastases (Figure 6(i**-j)**), and the protein level of ICAM1 was positively associated with ALPK1 expression (Supplementary Figure 5i). In addition, we followed up patients in Cohort 2 and analyzed the relevance of ALPK1 and ICAM1 expression to patient outcomes. Kaplan-Meier analysis showed that high expression of ALPK1 or ICAM1 was significantly associated with a shorter overall survival time of CRC patients (Figure 6(k**-l)**).

To further determine the role of *F.* *nucleatum* in CRC metastasis, we quantified the relative abundance of *F.* *nucleatum* in 32 fecal samples from CRC patients with positive lymph nodes metastasis (N1+N2) and 40 fecal samples without lymph nodes metastasis (N0) (Cohort 3). As expected, the level of *F.* *nucleatum* was specifically much higher in CRC patients with positive lymph nodes metastasis (Figure 6(m)). Meanwhile, there was an enrichment of *F.* *nucleatum* in the feces of CRC patients compared with the normal controls (Supplementary Figure 5j).

Taken together, our results indicate that the overabundance of *F.* *nucleatum* is associated with high expressions of ALPK1 and ICAM1 in CRC tissues. Furthermore, patients with high levels of *F.* *nucleatum*, ALPK1 or ICAM1 have a higher risk for poor clinical outcomes, which has important implications for their clinical management.

## Discussion

**Figure 7.**
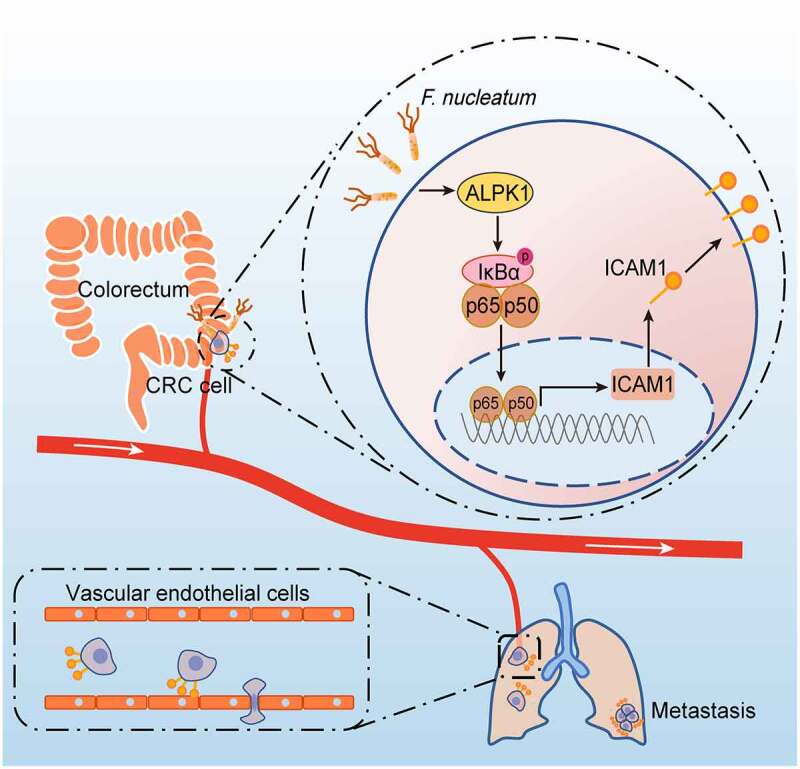
**Schematic diagram of *F. nucleatum*-induced ALPK1/NF-κB/ICAM1 axis regulating the metastasis of CRC.** The high abundance of *F.* *nucleatum* in gut activates NF-κB signaling of CRC cells through a newly identified pattern recognition receptor ALPK1, thereby promotes the expression of ICAM1, which is critical for *F.* *nucleatum*-induced CRC cell-endothelial cell adhesion, extravasation and metastasis.

In this study, we for the first time reveal that *F.* *nucleatum* enhances the intercellular adhesion ability of CRC cells, promotes CRC cells adhesion to endothelial cells, facilitates extravasation and metastasis, providing a new microbiota-host interaction model for controlling CRC metastasis (Figure 7). Mechanistically, *F.* *nucleatum* treatment activates NF-κB signaling of CRC cells through a newly identified pattern recognition receptor ALPK1, thereby promotes the expression of ICAM1, which is critical for *F.* *nucleatum*-induced CRC cell-endothelial cell adhesion and metastasis.

Metagenomic and transcriptomic analyses have revealed that gut microbiota, such as *Fusobacterium nucleatum*,^17^ *Akkermansia muciniphila*^31^ and *Streptococcus thermophilus*^32^ were involved in CRC development. Recently, the presence of *F.* *nucleatum* was detected in metastases and it may promote colorectal cancer metastasis. Several research groups have reported that *F.* *nucleatum* promotes colorectal cancer metastasis through the activation of *KRT7-AS*,^26^ the upregulation of CARD3 to activate autophagy,^33^ the induction of IL-8 and CXCL1^21^ or exosomes derived from *F.* *nucleatum*-infected colorectal cancer cells.^34^ However, tumor metastasis is an extremely complex process, including cancer cells detaching from the primary tumor, entering the blood circulation, adhering to endothelial cells and extravasation, reaching the distal part to form visible metastases.^5^ The direct interaction between tumor cells and endothelial cells is a crucial step for trans-endothelial invasion and colonization. Modulation of the expression of adhesion-related molecules may be an effective strategy for improving the prognosis of CRC. Our present study for the first time revealed that *F.* *nucleatum* enhanced the adhesion of CRC cells to endothelial cells *in* *vitro* and *in* *vivo*, which is a *F.* *nucleatum*-induced novel crosstalk between *F.* *nucleatum* and CRC cell-endothelial cell adhesion in promoting CRC metastasis.

In this study, we analyzed the mechanisms by which *F.* *nucleatum* induced the enhancement of CRC cell-endothelial cell adhesion. We identified ICAM1 overlapping between cell adhesion-related genes and *F.* *nucleatum*-upregulated genes in our RNA-sequencing results, as a direct downstream target of *F.* *nucleatum*. ICAM1, a cell surface adhesion molecule, could promote pancreatic ductal adenocarcinoma,^35^ hepatocellular carcinoma^36^ and colorectal cancer^37^ development. We found that the downregulation of ICAM1 decreased the promotion of *F.* *nucleatum*-induced CRC cell-endothelial cell adhesion and metastasis *in* *vitro* and *in* *vivo*.

Furthermore, we attempted to reveal the mechanisms for the regulation of ICAM1 by *F.* *nucleatum*. Our KEGG analysis based on RNA-sequencing results demonstrated that NF-κB pathway was activated by *F.* *nucleatum* treatment. Consistently, several other studies also confirmed the activation of NF-κB pathway in *F.* *nucleatum*-infected cells.^17,26,38,39^ In addition, the recognition of TLR4 on the surfaces of CRC cells was suggested to be involved in the sustained activation of NF-κB signaling pathway.^17^ However, *F.* *nucleatum* was reported to adhere to and invade epithelial cells via its virulence factors such as FadA,^39^ Fap2^40^ and FomA.^41^ Moreover, a recent study confirmed the invasive potential of *F.* *nucleatum* into HCT116 cells by fluorescence microscopy and flow cytometry,^21^ suggesting that there may exist other ways to activate NF-κB pathway. Intriguingly, a recent study identified ALPK1 as a key mediator of microbiota-induced NF-κB activation, which is a member of the atypical kinase family alpha kinases that recognize phosphorylation sites within α helices.^42,43^ It is generally acknowledged that ALPK1 accepted the signal of ADP-Hep present in Gram-negative bacterium.^23^ Recognition of ADP-Hep by ALPK1 represents a generic form of innate sensing, which mediates immune responses to diverse bacterial pathogens. Here, we found that *F.* *nucleatum* treatment led to an induction of ALPK1 and resulted in the sustained activation of NF-κB signaling, and silencing of ALPK1 inhibited NF-κB activation and ICAM1 upregulation by *F.* *nucleatum* infection. In addition, our results suggested that *F.* *nucleatum* exhibited a dramatic activation on ALPK1/NF-κB/ICAM1 pathway compared with other Gram-negative bacteria. ALPK1/NF-κB/ICAM1 is relatively a specific pathway for *F.* *nucleatum* infection. However, we did not dissect the detail mechanisms by which *F.* *nucleatum* activated ALPK1 pathway. Several research groups reported a similar manner that *Helicobacter pylori* activated ALPK1/NF-κB axis via the type-IV secretion system (T4SS).^42,44–46^ It will be of great interest to elucidate how *F.* *nucleatum* stimulates the expression of ALPK1 in CRC cells and further influences NF-κB pathway.

In addition, we found that the abundance of *F.* *nucleatum* and ICAM1/ALPK1 were highly enriched in CRC patients with metastasis. Moreover, the relative abundance of *F.* *nucleatum* was positively correlated with ICAM1/ALPK1 levels in CRC tissues, and the high expression of ICAM1/ALPK1 was significantly associated with a shorter overall survival time of CRC patients. Recent studies have reported that *F.* *nucleatum* promotes colorectal carcinogenesis by modulating Wnt/β-catenin pathway,^34,39,47^ NF-κB pathway,^17,26,39^ autophagy,^18,33^ glucose metabolism,^48^ etc. ALPK1/NF-κB/ICAM1 axis could, at least in part, explain how *F.* *nucleatum* treatment promotes CRC metastasis. However, it is possible that other mechanisms may be involved in the *F.* *nucleatum*-induced interactions between CRC cells and endothelial cells, and subsequent CRC metastasis. As the amount of *F.* *nucleatum* is associated with the risk of CRC metastasis, the measurement of *F.* *nucleatum* abundance may be an effective approach to predict patient outcome. Furthermore, our findings support the hypothesis that targeting *F.* *nucleatum*, host epithelial ICAM1 expression and/or ALPK1 may provide a means to block *F.* *nucleatum*-induced CRC metastasis and create diagnostic opportunities.

In summary, our current findings provide important insights into the molecular mechanisms underlying the regulation of an intercellular molecule, ICAM1, by *F.* *nucleatum* in CRC metastasis. *F.* *nucleatum* induces NF-κB pathway through ALPK1 activation, resulting in the upregulation of ICAM1 and enhanced CRC cell-endothelial cell adhesion and metastasis. Since the compelling evidences *in* *vitro* and *in* *vivo* highlight the emergence of *F.* *nucleatum*, ICAM1 or ALPK1 as pro-metastatic roles in CRC patients, a potential therapeutic strategy targeting *F.* *nucleatum*, ICAM1 and/or ALPK1 could be well-utilized.

## Methods

### Cell culture

Human colorectal cancer cell lines (LoVo, HCT116) and Human Umbilical Vein Endothelial Cells (HUVECs) were purchased from American Type Culture Collection (ATCC). HCT116 cultured in Maccoy 5A (Genom, China), LoVo cultured in F-12 K (Genom, China) and HUVECs cultured in high-glucose DMEM (GIBCO, China) were supplemented with 10% FBS (Sijiqing, China) at 37°C in a humidified 5% CO_2_ atmosphere.

### Bacterial strains and culture conditions

*F.* *nucleatum*, *A.* *muciniphila* and *B.* *adolescentis* were purchased from American Type Culture Collection (ATCC, *F.* *nucleatum* strain 25586 and 10953, *A.* *muciniphila* BAA-835 and *B.* *adolescentis* strain 15703). *F.* *nucleatum* was grown in Columbia blood agar (Comagal, China) in an anaerobic glove box at 37°C. *A.* *muciniphila* in Brain Heart Infusion (BD Difco, USA) supplemented with 0.05% mucin Type II (Sigma-Aldrich, USA), *B.* *adolescentis* in Reinforced Clostridium Medium (BD Difco, USA) were cultured under an atmosphere of 10% H_2_, 10% CO_2_, and 80% N_2_ in an AW500SG anaerobic workstation (ELECTROTEK) at 37°C. The *E.* *coli* strain DH5α (Takara, Japan) was cultured in Luria-Bertani medium overnight at 37°C.

### Human specimens

A total of 98 paired fresh tumor and adjacent non-tumor tissues were obtained from patients with primary CRC who did not receive preoperative anti-tumor treatment from Sir Run Run Shaw Hospital of Zhejiang University (Hangzhou, China). Fecal samples were collected from 72 CRC patients and 66 healthy people. All samples were frozen in liquid nitrogen until use. Another clinical tissue array cohort with 93 paired tumor and adjacent non-tumor tissues with or without liver metastasis were obtained from The Second Affiliated Hospital of Zhejiang University (Hangzhou, China).

### Patient-derived xenograft (PDX)

CRC surgical specimen was physically separated into small pieces (2–3 mm) and subcutaneously transplanted into nude mice. The xenografts were resected from mice and cut into similarly sized PDX tissues. Then the obtained PDX tumor tissues were randomly divided into two groups, and treated with PBS or *F.* *nucleatum* separately at a multiplicity of infection (MOI) of 100:1. Total RNAs were extracted from the PDX tissues by Trizol reagent (Invitrogen, USA) following the manufacturer’s instructions for subsequent experiments.

### Adhesion assay

Adhesion assay was performed as described previously.^49^ Briefly, HUVECs were seeded on 96-well plates and grown overnight to reach full confluence. 5 × 10^4^ GFP-labeled HCT116 cells pretreated with *F.* *nucleatum* were washed with PBS for three times, and re-suspended in serum-free culture medium to add onto the monolayer HUVECs and co-cultured at 37°C for 15 minutes. After washing by PBS softly for three times to remove the non-adherent cells, the adherent tumor cells on the monolayers were observed and imaged with a fluorescence microscope. Each experiment was analyzed in triplicate.

### Migration assay

CRC cells with the indicated treatment were incubated with *F.* *nucleatum* (MOI of 100:1) for 6 hours, and migration assay was performed as previously described.^50^ The migrated cells were observed and photographed under inverted microscope at 18 or 24 hours. Each experiment was analyzed in triplicate.

### Animal assays

SCID mice (6–8 weeks old), BALB/c nude mice (6–8 weeks old) and C57BL/6 mice (5–6 weeks old, 6–8 weeks old) were used for indicated studies, cultured in specific-pathogen-free (SPF) facilities.

For cancer cell extravasation model, GFP-labeled HCT116 cells pretreated with PBS or *F.* *nucleatum* (MOI of 100:1) were intravenously injected into SCID mice (2 × 10^6^ cells/per mouse). 24 hours after inoculation, the lungs were perfused with PBS by using gravity pressure through the heart. When the effluent became clear, the lungs were resected and the colonized HCT116 cells were measured by immunofluorescence assay, in which lung sections were stained for CD31 (red) as previously reported.^35,51^

For pulmonary metastasis model, HCT116 control cells or ICAM1-knockdown HCT116 cells were incubated with PBS or *F.* *nucleatum* (MOI of 100:1) for 24 hours and tail-vein injected into BALB/c nude mice (2 × 10^6^ cells/per mouse). The lungs were surgically excised after 8 weeks, and the gross images and H&E staining of lung tissue sections were used to evaluate tumor metastasis.

For *F. nucleatum*-treatment C57BL/6 mice model, PBS-resuspended 10^9^ colony-forming units (CFU) *F.* *nucleatum* or PBS were administrated into C57BL/6 mice (5–6 weeks old) every day, which were fed with 2 mg/ml streptomycin in the drinking water for 3 days beforehand to ensure the consistency of regular microbiota and facilitate *F.* *nucleatum* colonization as previously described.^17^ After 15 days, mice were sacrificed and the colons of mice were surgically excised for further analysis.

For AOM/DSS-induced CRC model, C57BL/6 mice (6–8 weeks old) were given one single intraperitoneal injection of carcinogen azoxymethane (AOM, Sigma) at 10 mg/kg body weight following by 5 successive days of 2.5% dextran sodium sulfate (DSS) in the drinking water, and then were given regular drinking water for 2 weeks. This cycle was then repeated for twice. Mice in the *F.* *nucleatum* and *E.* *coli* groups were administrated with 10^9^ CFU *F.* *nucleatum* or *E.* *coli* suspended in 300 μL sterile anaerobic PBS every 2 days, and the control group was administered with PBS. After 4 months, mice were sacrificed and the colons were surgically excised for further analysis.

### RNA sequencing

For RNA sequencing, HCT116 cells were co-cultured with PBS or *F.* *nucleatum* (MOI of 100:1) for 6 hours. Total RNA was isolated from indicated cells by Trizol reagent (Invitrogen, USA), and mRNA was purified using poly-T oligo-attached magnetic beads. Sequencing libraries were generated using NEBNext® UltraTM RNA Library Prep Kit for Illumina® (NEB, USA) following manufacturer’s recommendations. 150 bp Paired-end libraries were sequenced by Illumina PE150 platform. Paired-end clean reads were aligned to the human genome version hg19 using Hisat2 v2.0.5. Differential expression analysis of two groups was performed using the DESeq2. P value < .05 and | log_2_ (fold change) | > 1 were considered as significant.

### RNA extraction and quantitative RT-PCR

Total RNAs were extracted from CRC cell lines or tissue samples using Trizol reagent (Invitrogen, USA), and cDNAs were reversed by Evo M-MLV RT Kit (Accurate Biology, China) according to the manufacturer’s instructions. Quantitative RT-PCR analysis was performed in triplicate in ROCHE LightCycler480 System (Rotor gene 6000 Software, Sydney, Australia). Each reaction was tested in triplicate in 10 μl reaction system containing SYBR Premix Ex Taq (Takara, Japan), primers and template cDNA. Relative abundance was calculated by -ΔCt method as previously described.^27,52^ *GAPDH* or *ACTIN* were served as an internal reference gene. The following primer sets were used:

Human *ICAM1*: Forward: 5ʹ-CACCCTAGAGCCAAGGTGAC-3ʹ,

Reverse: 5ʹ-GGGCCATACAGGACACGAAG-3ʹ;

Human *ALPK1*: Forward: 5ʹ-TGACCACCATTTGCTGTCC-3ʹ,

Reverse: 5ʹ-ACGTGCCACGGATATTCAC-3ʹ

Human *GAPDH*: Forward: 5ʹ-GGAGCGAGATCCCTCCAAAAT-3ʹ,

Reverse: 5ʹ-GGCTGTTGTCATACTTCTCATGG-3ʹ

Mouse *ALPK1*: Forward: 5ʹ-CGCTATTGTCTTCTTGATGGACC-3ʹ

Reverse: 5ʹ-TTGACGGAGACTCGGGCTT-3ʹ;

Mouse *ICAM1*: Forward: 5ʹ-CACCCCGCAGGTCCAATTC-3ʹ,

Reverse: 5ʹ-TCCAGCCGAGGACCATACAG-3ʹ;

Mouse *ACTIN*: Forward: 5ʹ-AGCCATGTACGTAGCCATCC-3ʹ

Reverse: 5ʹ-CTCTCAGCTGTGGTGGTGAA-3ʹ.

### DNA extraction and *F.* *nucleatum* quantification

Genomic DNAs were extracted from clinical or mice tissues by QIAamp DNA Mini Kit (Qiagen, Germany) according to the manufacturer’s instructions. Genomic DNAs were extracted from clinical stool samples with TIANamp Stool DNA Kit (Tiangen, China). Genomic DNAs from each specimen were subjected to quantitative RT-PCR analysis to determine the relative abundance of *F.* *nucleatum* as described above. *Universal Eubacteria 16S* was used as an internal reference gene for stool samples. The *PGT* gene was used as an internal control for tissue samples as previously described.^27^ The following primer sets were used:

*F.* *nucleatum*: Forward: 5ʹ-CGGGTGAGTAACGCGTAAAG-3ʹ,

Reverse: 5ʹ-GCATTCGTTTCCAAATGTTGTCC-3ʹ;

*PGT*: Forward: 5ʹ-ATCCCCAAAGCACCTGGTTT-3ʹ,

Reverse: 5ʹ-AGAGGCCAAGATAGTCCTGGTAA-3ʹ;

*Universal Eubacteria 16S*: Forward: 5ʹ-CGGCAACGAGCGCAACCC-3ʹ,

Reverse: 5ʹ-CCATTGTAGCACGTGTGTAGCC-3ʹ.

### Immunohistochemistry (IHC) and Western blot analysis

IHC staining was performed as previously described.^53^ Tissue microarrays were scanned with a digital slide scanner (Pannoramic MIDI, 3D HISTECH) after staining and processed with Pannoramic viewer software. Intensity of staining in cells was automatic calculated by Quant center software. Histochemistry score (H-score) was acquired according to the formula: H-score = (percentage of weak intensity area ×1) + (percentage of moderate intensity area ×2) + (percentage of strong intensity area ×3). Western blot analysis was conducted as previously described.^54^ The following primary antibodies were used: ICAM1 (10831-1-AP, Proteintech), phospho-IκBα (#2859, CST), NF-κB p65 (#8242, CST), phospho-NF-κB p65 (#3033, CST), ALPK1 (GTX01077, GeneTex) and GAPDH (10494-1-AP, Proteintech).

### Immunofluorescence and confocal microscopy

Immunofluorescence staining was performed as previously described.^55^ Images were collected with a confocal microscope (LSM 880, Zeiss) and processed with ZEN image software. The following primary antibodies were used: CD31 (11265-1-AP, Proteintech), NF-κB p65 (#8242, CST).

### Cell transfection

The siRNAs were transfected into cells using Lipofectamine RNAiMAX (Thermo Fisher Scientific, USA) in opti-MEM (Genom, China) according to the manufacturer’s instructions. The following sequences of siRNAs were used:

siCtl: 5ʹ-UUCUCCGAACGUGUCACGUTT-3ʹ;

siICAM1-1: 5ʹ-CCUAUGGCAACGACUCCUUTT-3ʹ;

siICAM1-2: 5ʹ-CCUCAGCACGUACCUCUAUTT-3ʹ;

sip65-1: 5ʹ-CAGAUACAGACGAUCGUCATT-3ʹ;

sip65-2: 5ʹ-CCCUAUCCCUUUACGUCAUTT-3ʹ;

siALPK1-1: 5ʹ-GCAGGUUGGAUAAACUUAUTT-3ʹ;

siALPK1-2: 5’-CGCAGGCCCUACAUUUAAATT-3ʹ;

siTLR4: 5ʹ-GGGCUUAGAACAACUAGAATT-3ʹ;

siMYD88: 5ʹ-GGCAACUGGAACAGACAAATT-3ʹ.

### Plasmid construction and transfection

To generate shRNA constructs of ICAM1, oligos targeting mRNA of *ICAM1* were synthesized and cloned into lentiviral pLKO.1-puro vector (Addgene plasmid #8453). The full-length OFR of human ICAM1 (NM_000201.3) were prepared by cloning the cDNA from HEK293 cells into the pLVX-puro vector (Clontech #PT4002-5). Transient plasmid transfection was carried out using FuGENE HD transfection reagent (Promega, USA) according to the manufacturer’s instructions. The following primer sequences for vectors construction were used:

pLKO.1-sh ICAM1:

Forward: 5ʹ-CCGGGCTGACGTGTGCAGTAATACTTCTCGAGAAGTATTACTGCACACGTCAGCTTTTTTG-3ʹ,

Reverse: 5ʹ-AATTCAAAAAAGCTGACGTGTGCAGTAATACTTCTCGAGAAGTATTACTGCACACGTCAGC-3ʹ;

plvx- ICAM1-FL:

Forward: 5ʹ-ATGGCTCCCAGCAGCCCCCGG-3ʹ,

Reverse: 5ʹ-TCAGGGAGGCGTGGCTTGTGT GTTCG-3ʹ.

### Lentivirus production, precipitation and infection

To generate cells stably expressing short-hairpin RNAs (shRNAs), lentivirus production, precipitation and infection were performed as previously described.^56^

### Statistical analysis

Statistical analysis was performed using the GraphPad Prism 7.0 software. Data were analyzed with paired or unpaired Student’s t test, Mann-Whitney test, linear regression or Log-rank test as showed in figure legends. P value less than 0.05 was considered statistically significant.

## Supplementary Material

Supplemental Material

## Data Availability

The accession number for the data for RNA-sequencing reported in this study is NCBI GEO: GSE171611.

## Abbreviations

CRC: colorectal cancer; *F.* *nucleatum*: *Fusobacterium nucleatum*; *E.* *coli*: *Escherichia coli*; *A.* *muciniphila*: *Akkermansia muciniphila*; *B.* *adolescentis*: *Bifidobacterium adolescentis*; PBS: phosphate buffer saline; HUVECs: Human Umbilical Vein Endothelial Cells; PDX: patient-derived xenograft; MOI: multiplicity of infection; SPF: specific-pathogen-free; CFU: colony-forming units; NES: Normalized Enrichment Score; TCGA: The Cancer Genome Atlas; ATCC: American Type Culture Collection.

## Study approval

All animal experiments used in this study were approved by the Institutional Animal Care and Use Committee of Zhejiang University (SRRSH202102222). The collection of human samples was approved by the ethics committee of Sir Run Run Shaw Hospital and The Second Affiliated Hospital of Zhejiang University with informed written consent obtained from all participants (20180226-80, 2020-446).
