# Supplementary figures and images for: Fusobacterium nucleatum promotes colorectal cancer cells adhesion to endothelial cells and facilitates extravasation and metastasis by inducing ALPK1/NF-κB/ICAM1 axis
Source: Gut Microbes. 2022 Feb 27;14(1):2038852. doi: 10.1080/19490976.2022.2038852 (PMC8890384; doi:10.1080/19490976.2022.2038852)

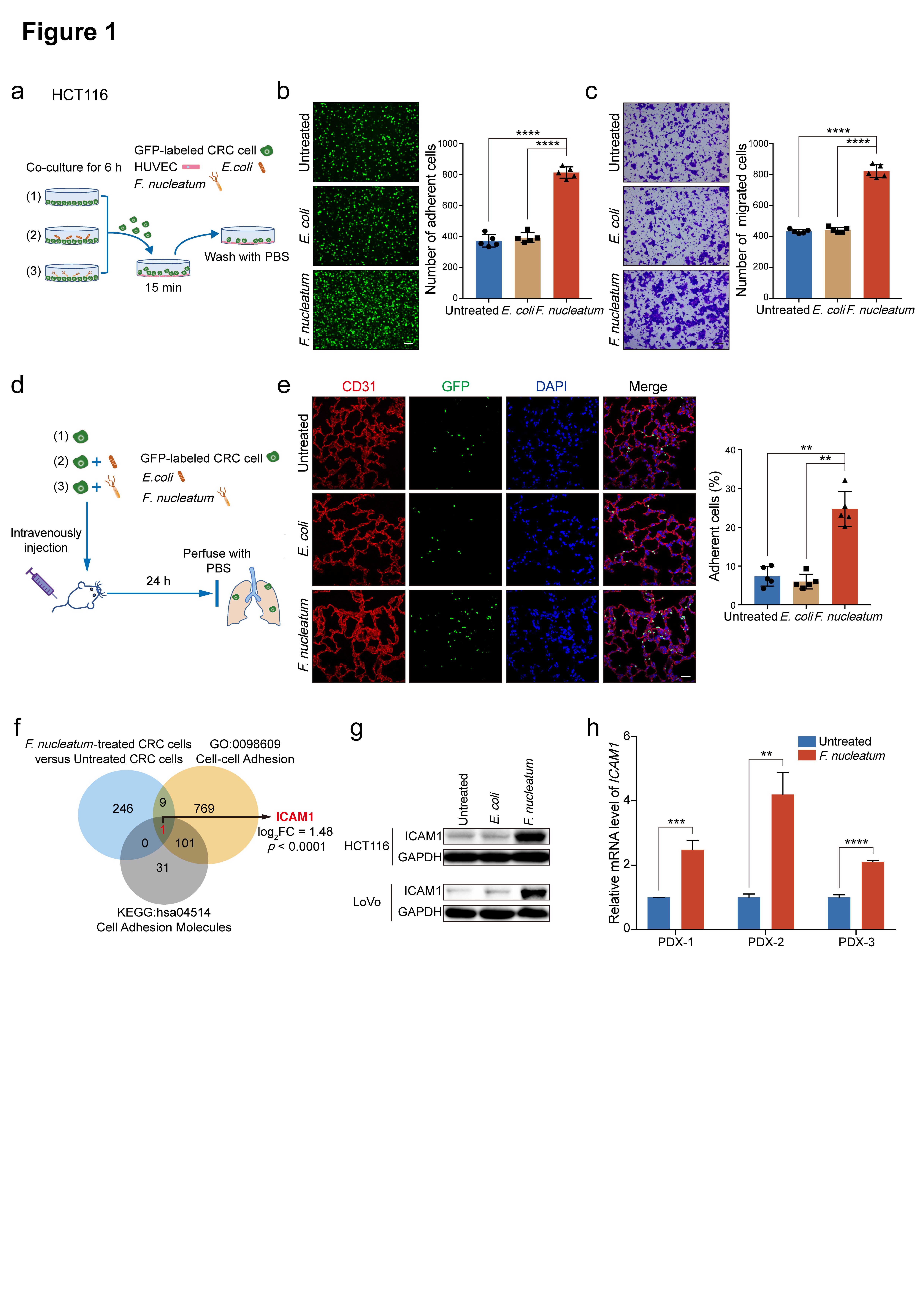

Supplement: Supplemental Material [file KGMI_A_2038852_SM2969.zip › supplementary/Supplementary Figure1 KGMI 20210488R1.tif]

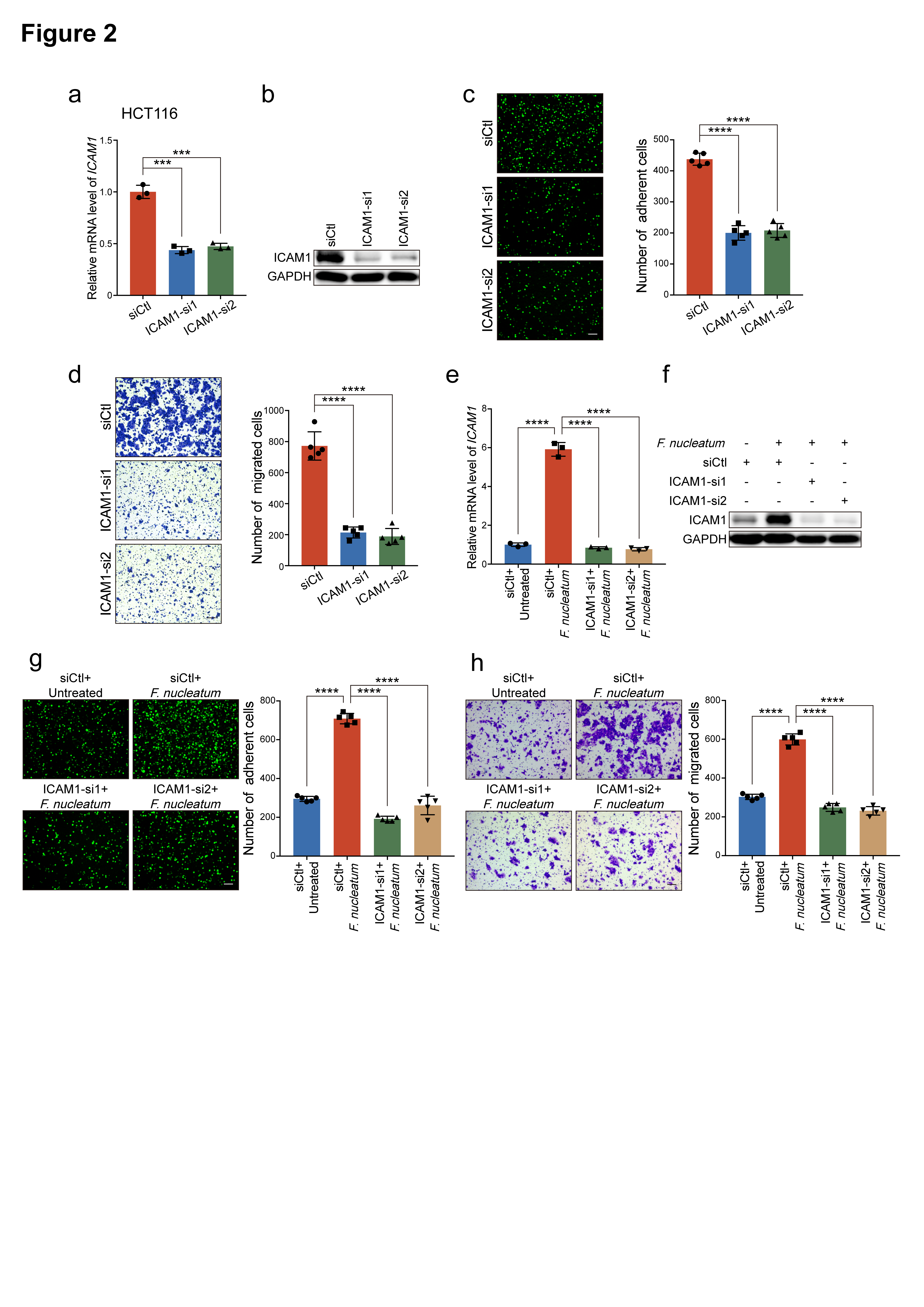

Supplement: Supplemental Material [file KGMI_A_2038852_SM2969.zip › supplementary/Supplementary Figure2 KGMI 20210488R1.tif]

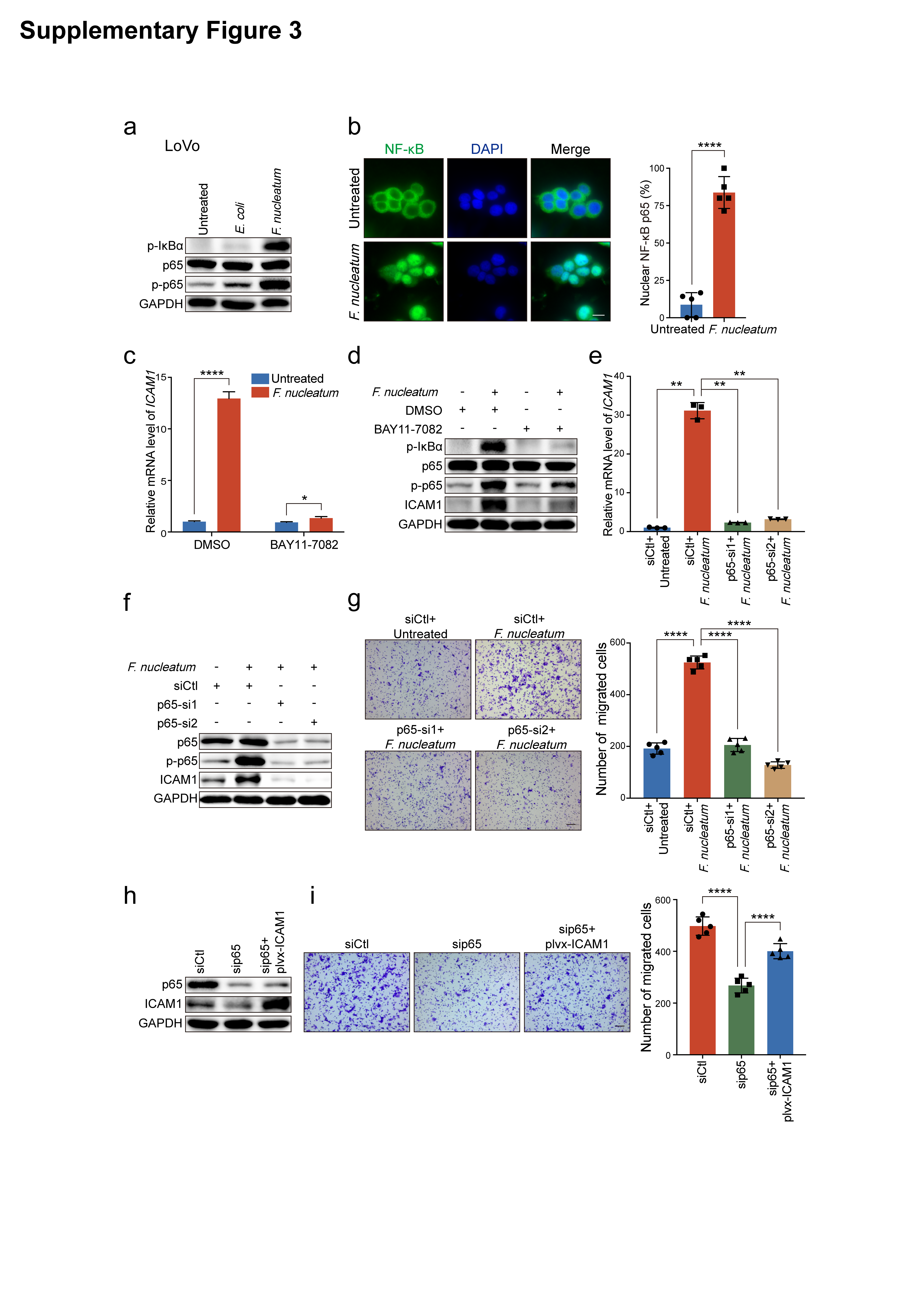

Supplement: Supplemental Material [file KGMI_A_2038852_SM2969.zip › supplementary/Supplementary Figure3 KGMI 20210488R1.tif]

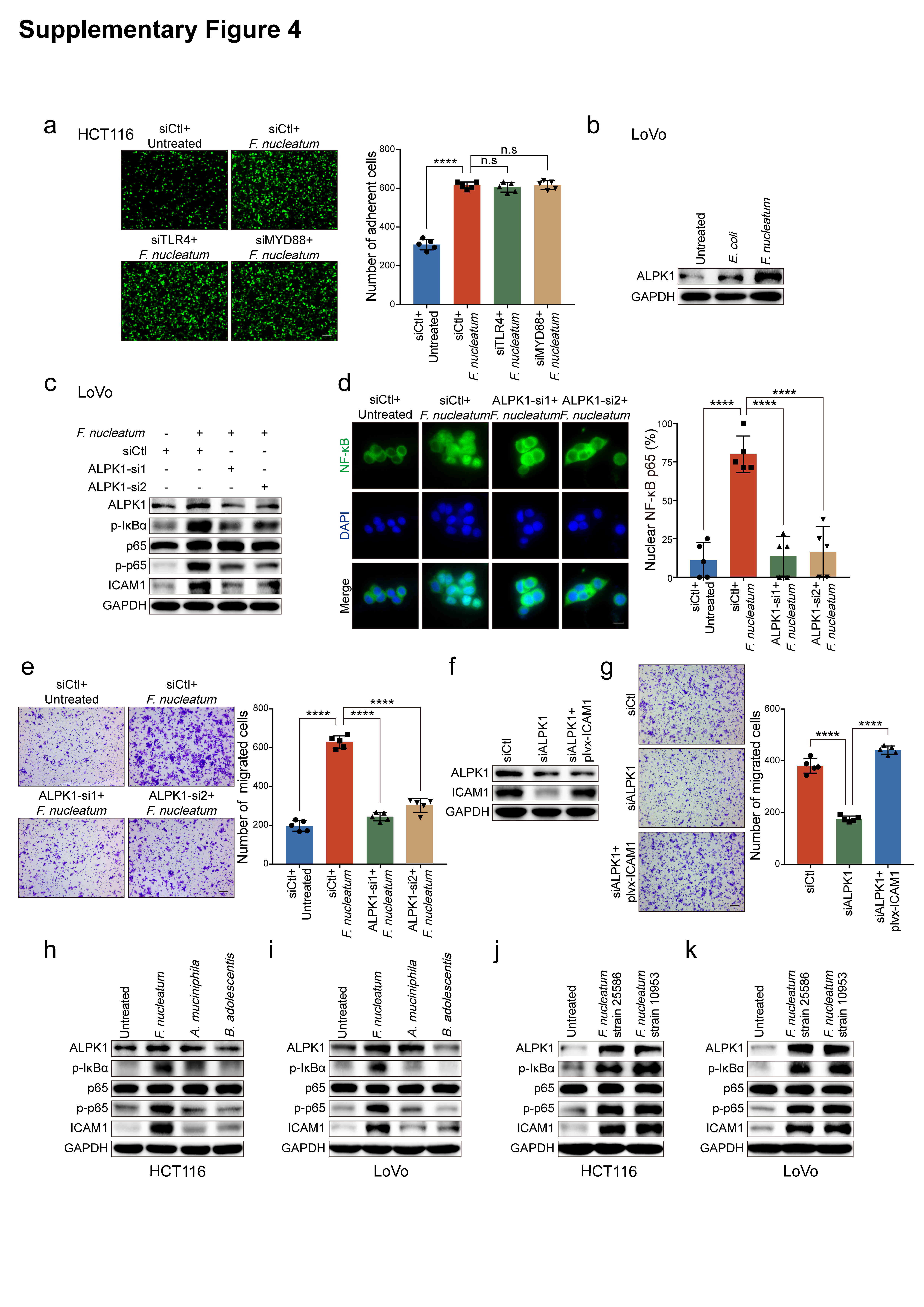

Supplement: Supplemental Material [file KGMI_A_2038852_SM2969.zip › supplementary/Supplementary Figure4 KGMI 20210488R1.tif]

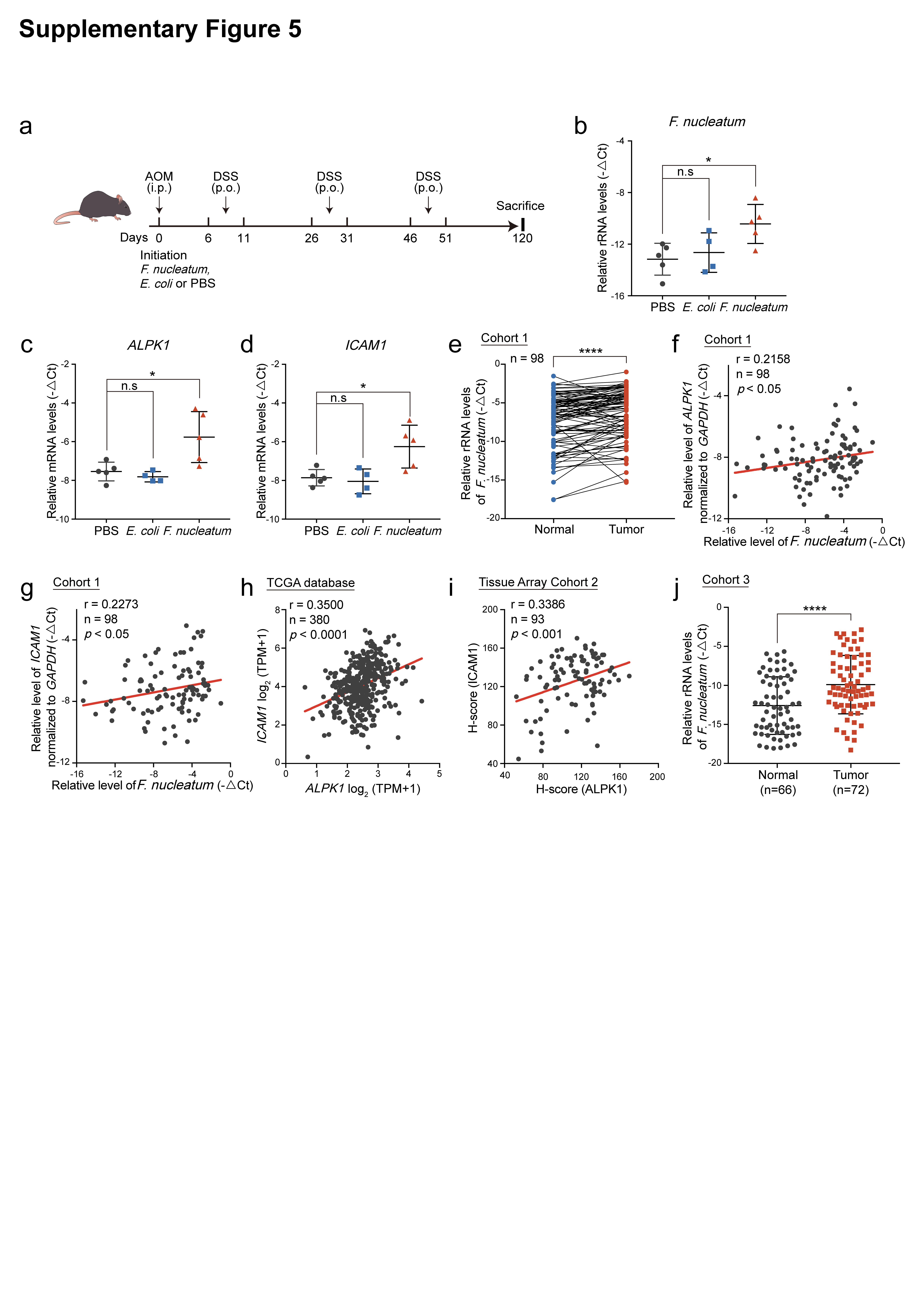

Supplement: Supplemental Material [file KGMI_A_2038852_SM2969.zip › supplementary/Supplementary Figure5 KGMI 20210488R1.tif]
